# Day and Night Changes of Cardiovascular Complexity: A Multi-Fractal Multi-Scale Analysis

**DOI:** 10.3390/e22040462

**Published:** 2020-04-18

**Authors:** Paolo Castiglioni, Stefano Omboni, Gianfranco Parati, Andrea Faini

**Affiliations:** 1 IRCCS Fondazione Don Carlo Gnocchi, 20148 Milan, Italy; 2 Italian Institute of Telemedicine, 21048 Solbiate Arno, Italy; stefano.omboni@iitelemed.org; 3 Scientific Research Department of Cardiology, Science and Technology Park for Biomedicine, Sechenov First Moscow State Medical University, 119991 Moscow, Russia; 4 Department of Medicine and Surgery, University of Milano-Bicocca, 20900 Monza, Italy; gianfranco.parati@unimib.it; 5 Istituto Auxologico Italiano, IRCCS, Department of Cardiovascular, Neural and Metabolic Sciences, S.Luca Hospital, 20149 Milan, Italy; a.faini@auxologico.it

**Keywords:** multifractality, multiscale complexity, detrended fluctuation analysis, heart rate, blood pressure, self-similarity

## Abstract

Recently, a multifractal-multiscale approach to detrended fluctuation analysis (DFA) was proposed to evaluate the cardiovascular fractal dynamics providing a surface of self-similarity coefficients α(*q*,τ), function of the scale τ, and moment order *q*. We hypothesize that this versatile DFA approach may reflect the cardiocirculatory adaptations in complexity and nonlinearity occurring during the day/night cycle. Our aim is, therefore, to quantify how α(*q*, τ) surfaces of cardiovascular series differ between daytime and night-time. We estimated α(*q*,τ) with −5 ≤ *q* ≤ 5 and 8 ≤ τ ≤ 2048 s for heart rate and blood pressure beat-to-beat series over periods of few hours during daytime wake and night-time sleep in 14 healthy participants. From the α(*q*,τ) surfaces, we estimated short-term (<16 s) and long-term (from 16 to 512 s) multifractal coefficients. Generating phase-shuffled surrogate series, we evaluated short-term and long-term indices of nonlinearity for each *q*. We found a long-term night/day modulation of α(*q*,τ) between 128 and 256 s affecting heart rate and blood pressure similarly, and multifractal short-term modulations at *q* < 0 for the heart rate and at *q* > 0 for the blood pressure. Consistent nonlinearity appeared at the shorter scales at night excluding *q* = 2. Long-term circadian modulations of the heart rate DFA were previously associated with the cardiac vulnerability period and our results may improve the risk stratification indicating the more relevant α(*q*,τ) area reflecting this rhythm. Furthermore, nonlinear components in the nocturnal α(*q*,τ) at *q* ≠ 2 suggest that DFA may effectively integrate the linear spectral information with complexity-domain information, possibly improving the monitoring of cardiac interventions and protocols of rehabilitation medicine.

## 1. Introduction

Time-series complexity is common in physiology. In fact, physiological systems often exhibit fractal geometries and are composed of several elements interacting nonlinearly, which are both typical features of a complex system [1]. The cardiovascular system, in particular, can be described as a complex, dynamical system because it is composed of a fractal network of branching tubes, the vasculature, connecting individual vascular beds that interact with each other to harmonize globally the local needs of blood supply. The overall cardiovascular regulation modulates the local blood flows thanks to the integrative control of the autonomic nervous system operating through effectors and feedbacks (the baro- and chemoreflexes) with nonlinear elements. 

Complex dynamical systems are not characterized by an intrinsic time scale. This means that their derived time series may appear statistically self-similar when plotted at different scales. For this reason, the interest in methods that quantify self-similar (or fractal) properties of the cardiovascular dynamics is increasing. A very popular method is based on the detrended fluctuation analysis (DFA), which provides a self-similarity scale coefficient, α, directly related to the Hurst’s exponent [2]. When DFA was originally proposed for the analysis of heart rate variability, it described a bi-scale fractal model providing a short-term coefficient (α_1_) for scales shorter than 16 beats and a long-term coefficient (α_2_) for longer scales [3]. The original bi-scale method was then extended in two ways. One way was to provide a multiscale spectrum of self-similarity coefficients, a function of the scale *n* in beats, α(*n*) [4,5,6]. Another way was to provide a multifractal spectrum of self-similarity coefficients, a function of the moment order *q*, α(*q*) [7,8]. The multifractal spectrum includes *q* = 2—the second-order moment used in the original DFA method for monofractal series—and allows detecting multifractality when α(*q*) differs substantially between positive and negative *q* orders. The multiscale and the multifractal methods were finally combined in the multifractal-multiscale DFA, a versatile approach that describes multifractal structures localized over specific scales and that provides a surface of scale coefficients, α(*q,n*) [9]. Recent works demonstrated the capability of the multifractal-multiscale DFA of heart rate variability to classify different types of cardiac patients [10] and to describe alterations in the heart rate complexity due to an impaired integrative autonomic control in paraplegic individuals [11].

It is less clear, however, whether complexity methods based on DFA can quantify nonlinear components. In this regard, theoretical analyses affirm that the information on the Hurst’s exponent provided by the second-order moment DFA can be derived mathematically from the power spectrum, which is a linear method of analysis [12,13]. Actually, empirical quantifications of the degree of nonlinear information of the cardiovascular dynamics provided by the more advanced multifractal-multiscale approaches are missing.

In this work, we hypothesize that the versatile multifractal-multiscale DFA approach may reflect the cardio-circulatory adaptations in the overall complexity and, particularly, in the nonlinear dynamics of the cardiovascular time series that may occur during the day/night cycle. Circadian rhythms and differences in activity levels between daytime and night-time hours are expected to have a major influence on cardiovascular regulation. Knowing how this happens may help to better identify and interpret possible alterations associated with pathological conditions. In this regard, a description of the α(*q,n*) circadian modulations may be important in the rehabilitation medicine for correctly monitoring changes associated with treatments or the recovery from clinical interventions. To our knowledge, no studies addressed the quantification of the changes in the multifractal-multiscale DFA of heart rate variability associated with the day–night cycle. Furthermore, most of the studies on cardiovascular complexity are based on the analysis of heart rate variability only. This is due to the difficulty to better describe the status of the system by measuring other cardiovascular variables beat-by-beat in addition to the heart rate, as the systolic and the diastolic arterial blood pressure.

Therefore, our work aims to address the above open issues on cardiovascular complexity by quantifying the fractal dynamics of heart rate and blood pressure, the degree of nonlinearity, and possible night–day modulations of complexity. This will be done analyzing continuous 24-h blood pressure recordings and comparing self-similarity coefficients estimated by the multifractal-multiscale approach over daytime and night-time. In particular, we will define new indices of the degree of nonlinearity based on the multifractal-multiscale DFA to quantify the additional information provided by this complexity method compared to traditional spectral methods.

## 2. Materials and Methods 

### 2.1. Subjects and Data Collection

The study is based on a historic database of 24-h ambulatory intra-arterial blood pressure recordings obtained at the University Hospital of Milan (Ospedale Maggiore Policlinico, Milan, Italy), for the diagnosis of hypertension [14]. Recordings were performed between the 1980s and the 1990s when intermittent noninvasive arm devices were not still in use in the clinical practice. 

As inclusion criteria, we selected only adult (>18 yro) normotensive subjects in which the suspected hypertensive state was excluded after the clinical evaluation. Exclusion criteria were smoking; obesity; clinical or laboratory evidence of health abnormalities, like cardiovascular disease or diabetes; prior drug treatment for hypertension; any alteration in glucose metabolism or renal function; and administration of cardiovascular drugs in the 4 weeks preceding the recording. We also excluded blood pressure tracings of inadequate quality for a 24-h analysis. This led to selecting recordings of N = 14 normotensive subjects (3 females of which one in the childbearing age and 11 males) with age between 19 and 64 years.

Details of data collection are reported in [14]. Briefly, a catheter inserted into the radial artery of the non-dominant arm was connected to a transducing-perfusing unit secured to the thorax at the heart level. The blood pressure signal was stored on a magnetic tape recorder bound to the waist. During the recordings, the subjects were free to move within the hospital. Mealtimes and bedtimes were standardized. The blood pressure signal was digitized (170 Hz, 12 bits) and edited manually from movement artifacts, pulse pressure dampening, and premature beats. Each pulse wave was identified by a derivative-and-threshold algorithm [15]; systolic blood pressure (SBP) and diastolic blood pressure (DBP) were calculated for each pulse wave beat-by-beat. As suggested in [16], a parabolic interpolation refined the SBP fiducial point before calculating the inter-beat interval (IBI) as the interval between the times of occurrence of consecutive systolic peaks.

Two sub-periods were selected for the analysis after visual inspection of the tracings: the “Day” subperiod during daytime in the afternoon, when the subjects were not lying in bed and were free to perform normal daytime activities; the “Night” subperiod after 11 PM when the participants were asleep according to the schedule of the hospital. The selected segments had to be composed of at least 14,000 heartbeats, with a duration of at least 4 h during daytime and of at least 5 h during night-time, without evident nonstationarities. 

The study was carried out after having obtained informed consent from the participants in accordance with the 1975 Declaration of Helsinki and following the recommendations of the ethical committee of the Ospedale Maggiore Policlinico (Milan, Italy). 

### 2.2. Multifractal-Multiscale Detrended Fluctuation Analysis

We estimated the multifractal multiscale structure of the IBI, SBP, and DBP time series by the fast DFA algorithm available in [17]. Given the beat-by-beat series *x_i_* of length *L* beats, we calculated its cumulative sum, *y_i_*. We split *y_i_* into *M* maximally overlapped blocks of *n* beats (two consecutive blocks have *n*-1 beats in common). We detrended each block with least-square polynomial regression and calculated the variance of the residuals in each *k-th* block, σ^2^*_n_*(*k*). The variability function *F_q_*(*n*) is the *q*-th moment of σ^2^*_n_* [7]:

(1) $$\{\begin{array}{ll} F_{q}(n)={\left(\frac{1}{M}{\sum_{k=1}^{M}\left(\sigma_{n}^{2}\left(k\right)\right)}^{q/2}\right)}^{1/q} & \mathrm{for}\;q\neq 0\\ \;F_{q}(n)=e^{\frac{1}{2M}\sum_{k=1}^{M}\ln\left(\sigma_{n}^{2}\left(k\right)\right)} & \mathrm{for}\;q=0 \end{array}$$

**Figure 1:**
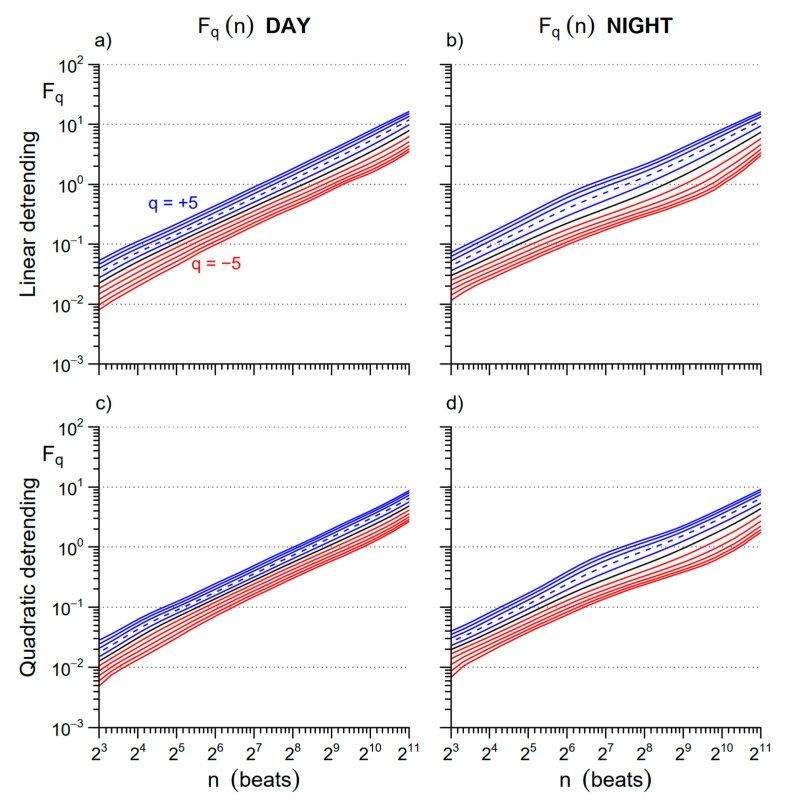
Multifractal variability functions *F_q_*(*n*) for inter-beat interval (IBI) with different orders of detrending polynomials: average over the group of participants. The *F_q_*(*n*) functions are plotted in blue for *q* > 0, in black for *q* = 0, and in red for *q* < 0; the dashed line is *q* = 2, second-order moment of the traditional monofractal detrended fluctuation analysis (DFA). Upper panels: *F_q_*(*n*) estimated with 1st order (linear) detrending during (**a**) Day and (**b**) Night. Lower panels: *F_q_*(*n*) with 2nd-order (quadratic) detrending during (**c**) Day and (**d**) Night.

We evaluated Equation (1) for *q* between −5 and +5 and block sizes *n* between 6 and *L*/4 beats. We evaluated the multifractal multiscale coefficients as a function of the beat-scale *n*, α_B_(*q*,*n*), calculating the derivative of log *F_q_*(*n*) vs. log *n* [17]. This was done for detrending polynomials of order 1 and 2 (see examples of the corresponding *F_q_*(*n*) estimates in Figure 1. Previous empirical analyses suggested that the second-order polynomial overfits block sizes shorter than 12 beats, but at the same time, it appears to more efficiently remove long-term trends [17,18,19]. Therefore, we estimated a single α_B_(*q*,*n*) function combining the estimates after detrending of order 1 and 2 with a weighted average which weights more the order one at the shorter scales as proposed in [17]. 

It should be noted that the parameter *q* in Equation (1) defines the moment order calculated for the variances of the residuals. In the traditional monofractal DFA, the variability function is defined as the root-mean-square of σ^2^*_n_*, which corresponds to the second-order moment, or *q* = 2. If the series is monofractal, all the moment orders *q* provide the same slope α. By contrast, for multifractal series positive moment order *q* weight more the contribution of the fractal components with greater amplitude, negative moment order *q* weight more the contribution of the fractal components with lower amplitude.

To compare Day and Night periods over the same temporal scales, in seconds, we mapped the scale units from number of beats, *n*, to time τ, in seconds, with the transformation

τ = *n* × μ_IBI_ (2)

with

(3) $$\mu_{\mathrm{IBI}}=\frac{1}{L}\sum_{i=1}^{L}IBI_{i}$$

the mean of the IBI values for all the *L* beats composing the whole time series, in seconds. The obtained coefficients expressed as a function of the time scale, α*(q,*τ*)*, were spline-interpolated over *τ* and resampled (2500 points evenly spaced over the logarithmic τ axis from 6 s to 3600 s) to have estimates at the same temporal scales for each recording. Realigning the DFA coefficients in this way allows properly comparing the same time scales between conditions in which the cardiovascular signals are sampled at different heart rates. We considered scales between 8 and 2048 s. The largest scale (τ = 2048 s) is estimated on more than seven independent blocks of data even in the case of the recording with the shortest duration: this assures sufficient stability of the estimate as shown empirically in previous validations [5,20]. Scales shorter than τ = 8 s were not considered because at negative *q* orders high levels of estimation bias may be present [17]. 

We introduced multifractal short-term and long-term coefficients to concisely describe the multifractal multiscale structure. This was done by averaging α(*q*,τ) over short scales, with 8 ≤ τ ≤ 16 s, and over long scales, with 16 < τ ≤ 512 s, obtaining the multifractal short-term coefficient α_S_(*q*) and long-term coefficient α_L_(*q*).

### 2.3. Nonlinearity Index 

For each series *j* we generated 100 Fourier phase-randomized series by shuffling the spectrum of the phases with the code available in [21]. This procedure removes possible nonlinear components in the dynamics of the original series, preserving its power spectrum and therefore the original first- and second-order moments [22]. Then, we calculated the multifractal multiscale coefficients of each of the 100 surrogates, α*^i,j^*(*q*,τ) with 1 ≤ *I* ≤ 100, to be compared with the coefficients of the original series *j*, α^O,*j*^(*q*,τ). For the comparison, we calculated π*^j^*(*q*,τ), defined at each *q* and τ as the percentile of the distribution of 100 surrogate α*^i,j^*(*q*,τ) coefficients in which was the original α^O,*j*^(*q*,τ) coefficient (to apply a 2-tail statistics, percentiles greater than 50% were transformed into their complement to 1 as in [23]). π*^j^*(*q*,τ) may range between 50% and 0%: the lower its value, the more significant the deviation of the original scale coefficient α^O,*j*^ from the distribution of the 100 surrogate coefficients α*^i^*^,*j*^. Large deviations from the surrogates distribution are suggestive of nonlinear components in the original series. Therefore, we defined a short-term nonlinearity index at each moment order *q*, *NL*_S_(*q*), by calculating the percentage of scales in the range 8 ≤ τ ≤ 16 s, where π*^j^*(*q*,τ) was ≤1%. Similarly, we calculated the percentage of scales with π*^j^(q,*τ*)* ≤ 1% for 16 < τ ≤ 512 s to define the long-term nonlinearity index *NL*_L_(*q*). Both *NL*_S_(*q*) and *NL*_L_(*q*) may range between 0% and 100%. Their higher values indicate moment orders *q* that better detect the presence of nonlinear components. 

### 2.4. Spectral Analysis

The IBI, SBP, and DBP beat-by-beat series were interpolated evenly at 5 Hz before spectral analysis. Power spectra were estimated by the Welch periodogram with 80% overlapped Hann data windows of 240 s length. The spectra were integrated over the very-low frequency (VLF, between 0.003 and 0.04 Hz), the low frequency (LF, between 0.04 and 0.15 Hz), and the high-frequency (HF, between 0.15 and 0.4 Hz) bands as indicated in the guidelines [16].

### 2.5. Statistical Analysis

The α(*q*,τ) coefficients of the *N* = 14 participants were compared between *Day* and *Night* at each τ and *q* by the Wilcoxon signed-rank test. The multifractal short- and long-term coefficients, α_S_(*q*) and α_L_(*q*), and nonlinearity indices, *NL*_S_(*q*) and *NL*_L_(*q*), were also compared between *Day* and *Night* at each *q* by the Wilcoxon signed-rank test. IBI, SBP, and DBP levels and power spectra were compared between *Day* and *Night* by the paired t-test, after log-transformation of the spectral indices to remove the skewness of their distribution [24]. The threshold for statistical significance was set at 5% with a two-sided alternative hypothesis. All the tests were performed with “R: A Language and Environment for Statistical Computing” software package (R Core Team, R Foundation for Statistical Computing, Vienna, Austria, 2019).

## 3. Results

### 3.1. Day vs. Night

The data segments selected for the analysis of the *Day* and *Night* periods were composed by a similar number of heartbeats: 20,779 (2744) beats during the *Day* and 20,329 (4059) beats during the *Night*, as average (SD) over the group. Means and spectral powers of the cardiovascular series are reported in Table 1. Because of the higher heart rate during the daytime, the segment duration was shorter in the *Day*, i.e., 4 h 30′ (30′), than in the *Night* period, i.e., 5 h 42′ (36′). For the same reason, the scale τ = 16 s that divides the α_S_(*q*) and α_L_(*q*) indices corresponds on average to 20.7 beats in the *Day* and 15.5 beats in the *Night* period, and the α_L_(*q*) upper scale at τ = 512 s corresponds to 661.2 beats and 495.3 beats in the *Day* and *Night* periods, respectively.

**Figure 2:**
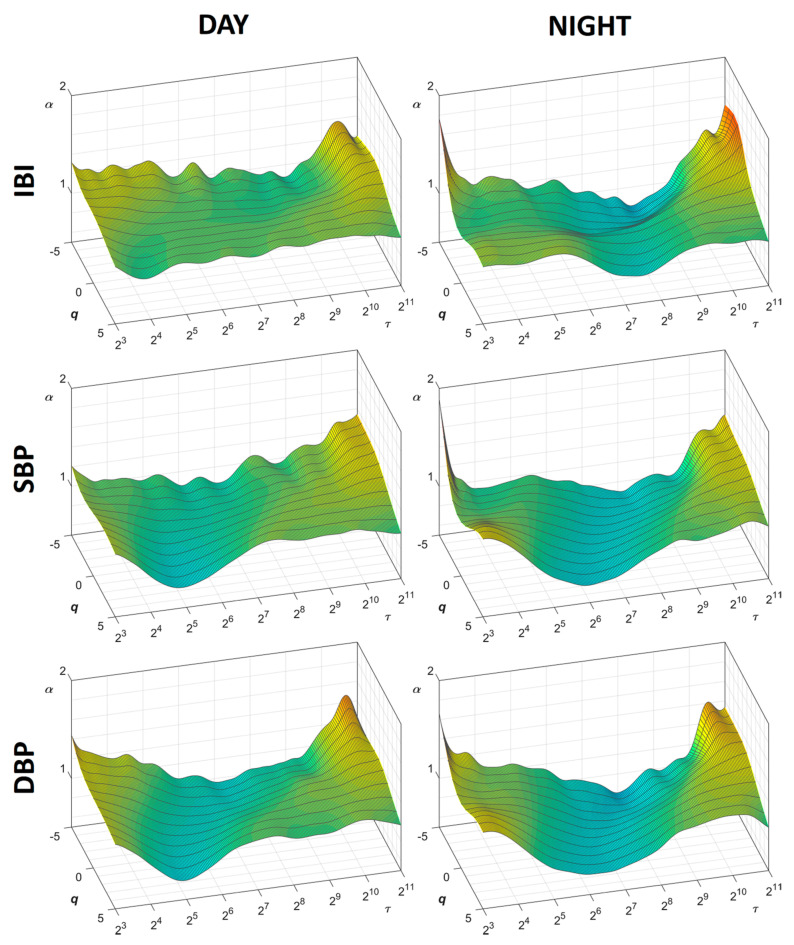
Surfaces of multifractal multiscale DFA coefficients, α(*q*,τ), during Day and Night periods. Average over 14 participants, for scales τ between 8 and 2048 s and moment orders *q* between −5 and +5; IBI = inter-beat-interval; SBP = systolic blood pressure; DBP = diastolic blood pressure.

Figure 2 shows the α(*q*,τ) surfaces for IBI, SBP, and DBP, separately, during *Day* and *Night* periods (average over the group of patients). The figure suggests the presence of structural differences between heart rate and blood pressure in their complex dynamics: during the daytime, these differences appear particularly clear between 16 and 256 s, where IBI appears characterized by a relatively flat surface at all *q* orders while SBP and DBP show a dip around τ = 32 s for positive *q* orders. 

Even more obvious is the difference between daytime and night-time in each cardiovascular series. The difference is particularly evident for the IBI surface of scale coefficients, which shows a marked decrease of the α coefficients at scales between 128 and 256 s during the night, more pronounced at negative *q* orders. Similar deflections appear to also characterize the surfaces of DFA scale coefficients of SBP and DBP. 

**Figure 3:**
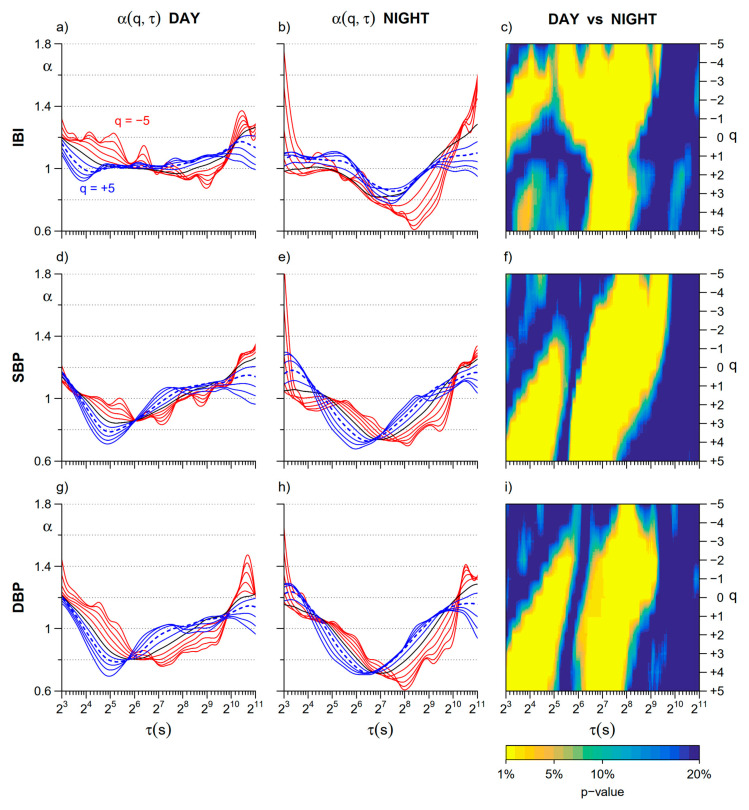
Day–Night comparison of cross sections of multifractal multiscale DFA coefficients. (**a**) Cross sections of α(*q*,τ) of IBI for scales τ between 8 and 2048 s and moment orders *q* between −5 and +5: average over the group of 14 participants in the *Day* subperiod; *q* < 0 in red, *q* > 0 in blue, *q* = 0 in black; the dotted line is α for *q* = 2 (second order moment of the monofractal DFA); (**b**) α(*q*,τ) of IBI as in panel (**a**) for the *Night* subperiod; (**c**) color map representing the statistical significance (*p* value) of the *Day* vs. *Night* comparison of IBI scale coefficients calculated at each τ and *q* after the Wilcoxon signed rank test; (**d**) α(*q*, τ) of SBP in the *Day* subperiod represented as in panel (**a**); (**e**) α(*q*, τ) of SBP in the *Night* subperiod represented as in panel (**a**); (**f**) color map of the *Day* vs. *Night* statistical significance for SBP scale coefficients; (**g**) α(*q*, τ) of DBP during *Day* represented as in panel (**a**); (**h**) α(*q*, τ) of DBP during *Night* represented as in panel (**a**); (**i**) color map of the *Day* vs. *Night* statistical significance for DBP coefficients.

Figure 3 compares *Day* and *Night* cross sections of the α(*q*,τ) surfaces at each moment order *q*. As to IBI, differences at scales shorter than 16 s regard two distinct *q*-τ areas. In the main area, centered at *q* = −2, α is lower at night, while in the secondary narrower area, centered at *q* = 4, α is greater at night. Remarkable *Day–Night* differences with lower α at night also regard scales between 128 and 256 s. They are evident at all the moment orders but extend over a larger range of scales τ for negative *q* values.

Similarly to IBI, also the α(*q*,τ) coefficients of SBP and DBP show a significant decrease at *Night* for scales between 128 and 256 s for all the *q* orders. Significant *Day–Night* differences with greater α at night also appear in blood pressure at scales shorter than 32 s, but, differently from IBI, the changes are significant for positive *q* only.

**Figure 4:**
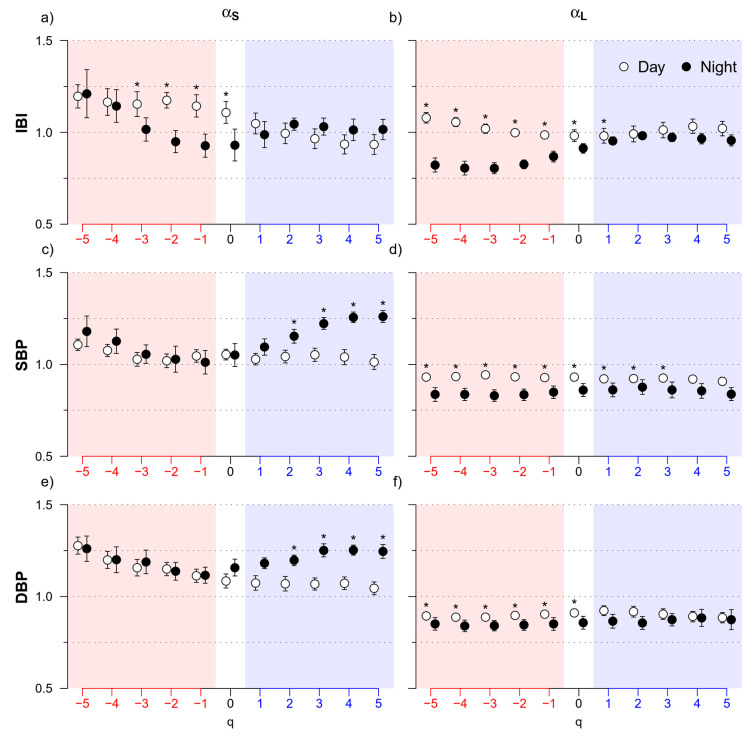
Day–Night comparison of multifractal short- and long-term coefficients. (**a**) Short-term coefficients, α_S_(*q*), for IBI in *Day* (open circles) and *Night* (solid circles) periods and for −5 ≤ *q* ≤ +5: median ±standard error of the median over N = 14 participants; the * indicates *Day* vs. *Night* differences significant at *p* < 0.05; (**b**) long-term coefficients, α_L_(*q*), of IBI represented as in panel (**a**); (**c**) short-term coefficients of SBP and (**d**) long-term coefficients of SBP, represented as in panel (**a**); (**e**) short-term coefficients and (**f**) long-term coefficients of DBP, represented as in panel (**a**).

The detailed representation of Figure 3 is summarized by the multifractal short- and long-term coefficients in Figure 4. The IBI multifractal short-term coefficient is significantly lower at night for −3 ≤ *q* ≤ 0, while the long-term coefficient is significantly lower at night for *q* ≤ 1. Moreover, the multifractal short-term coefficients of blood pressure are higher at night when *q* ≥ 2 (Figure 4c,e), while the long-term coefficients, as for IBI, are lower at night mainly for negative *q* (Figure 4d,f).

### 3.2. Nonlinearity

**Figure 5:**
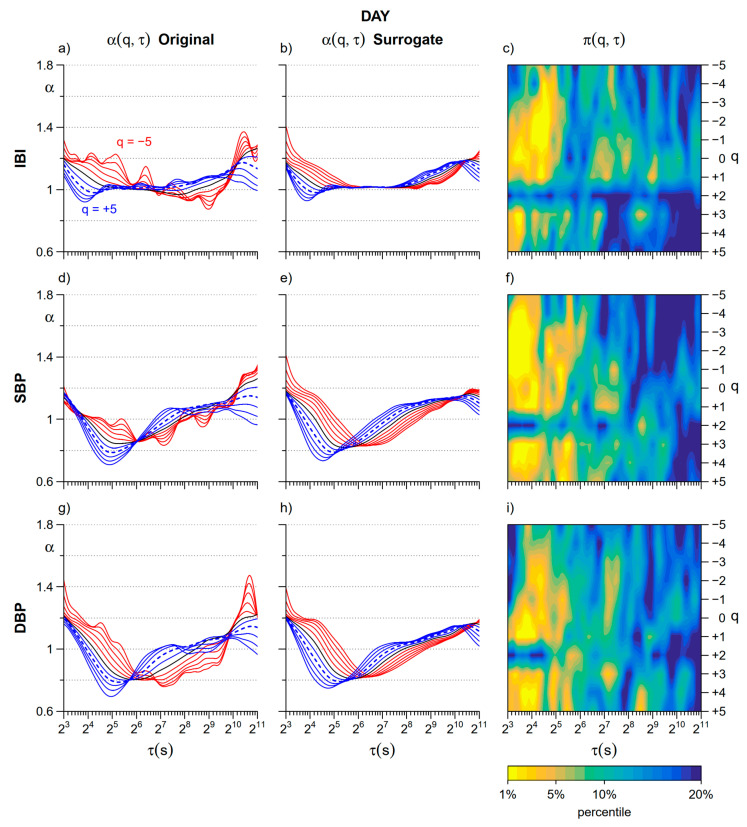
Assessment of nonlinearity during daytime. Upper panels refer to IBI: (**a**) α(*q*,τ) coefficients for the original series (average over N = 14 participants, see panel (**a**) for line colors); (**b**) α(*q*,τ) for the corresponding phase-randomized surrogate series; (**c**) color map of the percentile of the distribution of surrogate estimates in which is the original estimate (average over N = 14 participants). Mid panels refer to SBP: (**d**) α(*q*,τ) for the original series; (**e**) α(*q*,τ) for the corresponding surrogate series; (**f**) color map of percentiles. Lower panels refer to DBP: (**g**) α(*q*,τ) for the original series; (**h**) α(*q*,τ) for the corresponding surrogate series; (**i**) color map of percentiles.

Figure 5 illustrates the degree of nonlinearity detected comparing α(*q*,τ) of the original and surrogate series during the daytime. A common feature to heart rate and blood pressure is the evidence of nonlinear components at scales shorter than 64 s at all *q* but *q* = 2 (the moment order of the traditional monofractal DFA).

**Figure 6:**
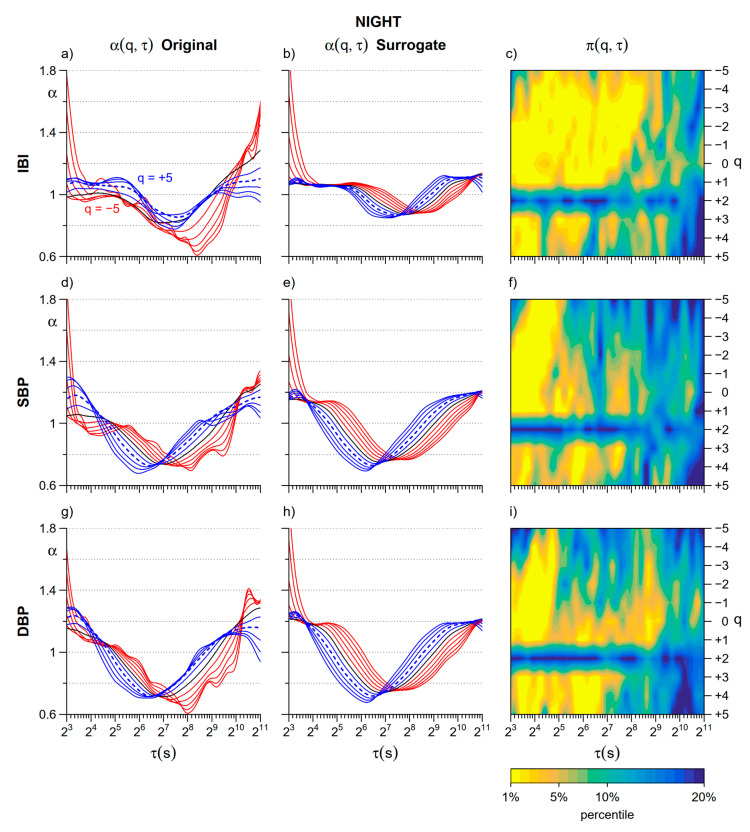
Assessment of nonlinearity during night-time. Upper panels refer to IBI: (**a**) *α*(*q*,*τ*) for the original series (average over N = 14 participants, see panel (**a**) for of line colors), (**b**) *α*(*q*,*τ*) for the corresponding phase-randomized surrogate series, and (**c**) color map of the percentile of the distribution of surrogate estimates in which is the original estimate (average over N = 14 participants). Mid panels refer to SBP: (**d**) *α*(*q*,*τ*) for the original series; (**e**) *α*(*q*,*τ*) for the surrogate series; (**f**) color map of percentiles. Lower panels refer to DBP: (**g**) *α*(*q*,*τ*) for the original series; (**h**) *α*(*q*,*τ*) for the surrogate series; (**i**) color map of percentiles.

At *Night* nonlinear components are more evident (Figure 6) and affect longer scales, particularly for IBI. Estimates at *q* = 2 appear to be linear also at night-time.

**Figure 7:**
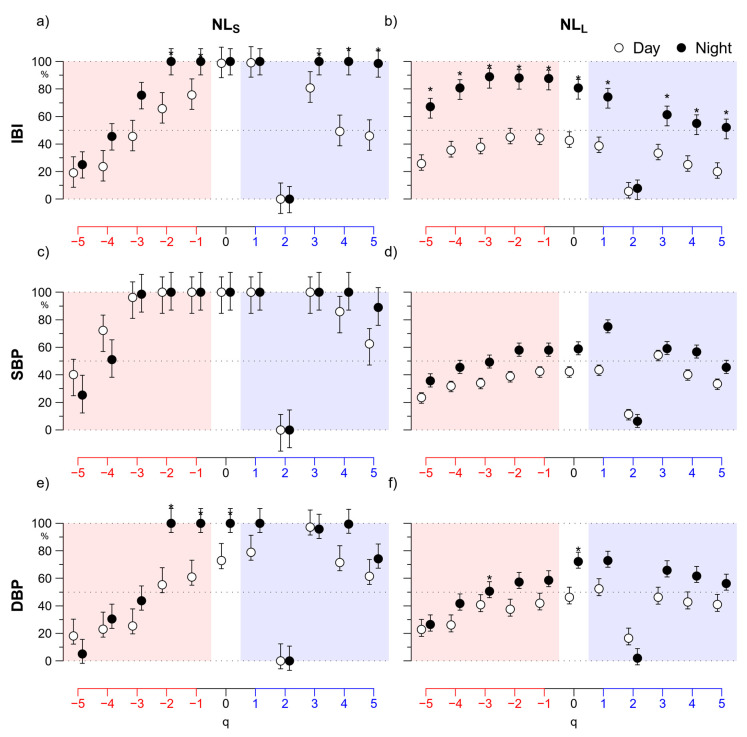
Day vs. Night comparison of short-term and long-term indices of nonlinearity. (**a**) Short-term index, *NL*_S_(*q*), for IBI in *Day* (open circles) and *Night* (solid circles) periods and for −5 ≤ *q* ≤ +5: median ±standard error of the median over N = 14 participants; the * indicates *Day* vs. *Night* differences significant at *p* < 0.05; (**b**) long-term nonlinearity index, *NL*_L_(*q*), of IBI; (**c**) short-term and (**d**) long-term nonlinearity index of SBP; (**e**) short-term and (**f**) long-term nonlinearity index of DBP.

Figure 7 summarizes these findings showing the short-term and the long-term nonlinearity indices, *NL*_S_(*q*) and *NL*_L_(*q*). The highest degree of nonlinearity is detected at *Night* by *NL*_S_(*q*), which is close to 100% for all the cardiovascular series between *q* = −2 and *q* = +4, with the notable exception of *q* = 2. In fact, at *q* = 2 *NL*_S_ falls to 0% for all the signals. *NL*_S_ tends to be higher at night with significant differences at some *q* < 0 for IBI and DBP and at *q* > 2 for IBI. Long-term nonlinear components are mainly present in IBI at night. In fact, *NL*_L_(*q*) of IBI is greater than 50% during night-time at all *q* but *q* = 2. Furthermore, it is significantly greater at night for all *q* ≠ 2. *NL*_L_ too is close to 0% at *q* = 2, both during *Day* and *Night*, for heart rate and blood pressure.

## 4. Discussion

This work compared patterns of blood pressure and heart rate complexity between daytime and night-time as assessed by the multifractal multiscale DFA approach. To our knowledge, this is the first study addressing night–day changes of multifractality in different cardiovascular signals and on a continuum spectrum of temporal scales. Our work revealed specific scales τ and specific fractal components (as identified by *q*) where the cardiovascular complexity differs between wake at daytime and sleep at night. Furthermore, it introduced new indices of nonlinearity which highlight the areas of the α(*q*,*τ*) surface that better reflect the nonlinear dynamics. A brief discussion of these points follows.

### 4.1. Day vs. Night

Mean levels and spectral powers of heart and blood pressure (Table 1) reflect the day–night changes reported previously [25,26], i.e., lower heart rate and blood pressure at night due to the lower levels of physical activity and to the lying position, which are associated with a higher cardiac vagal tone (HF power of IBI), a lower cardiac sympatho/vagal balance (LF/HF powers ratio of IBI), and a lower vascular sympathetic tone (LF power of SBP and DBP [27,28]). 

In addition to these known changes in heart rate and blood pressure mean levels and spectral powers, we reported clear changes in the α(*q*,*τ*) fractal structure. In IBI, the more evident change is the night decrease of coefficients around 128–256 s (Figure 4c). The decrease affects all the moment orders but it is amplified at negative *q* and thus the night/day modulation of the long-term multifractal index α_L_(*q*) is larger for *q* < 0 (Figure 4b). We may associate this night/day oscillation to an endogenous circadian rhythm previously described in the heart rate by a monofractal DFA exponent (i.e., for *q* = 2) estimated over scales between 20 and 400 beats [29]. This endogenous rhythm was hypothesized to contribute to the period of the cardiac vulnerability reported in epidemiological studies. Our work suggests that this night/day rhythm (1) is highlighted by a multifractal approach that assesses negative moment orders and (2) is better quantified in a narrower range of scales, between 128 s and 256 s. Therefore, our results may prove to be of clinical importance by allowing designing new tools for the complexity analysis of heart rate that better stratify the cardiovascular risk. Interestingly, our study also provides evidence that a night/day modulation with greater daytime values is present at the same scales in blood pressure too, suggesting that a common physiological mechanism is at the origin of the circadian oscillation in the heart rate and the blood pressure self-similarity coefficients. 

By contrast, night–day changes at shorter scales affect heart rate and blood pressure differently. While short-term coefficients of blood pressure are greater at night for moment orders *q* ≥ 2, the main modulation of short-term scales of heart rate consists of lower values at night for −3 ≤ *q* ≤ 0 (Figure 4a). Further studies controlling the effects of posture and physical activity are needed to understand the nature of so different night/day changes between heart rate and blood pressure.

**Figure 8:**
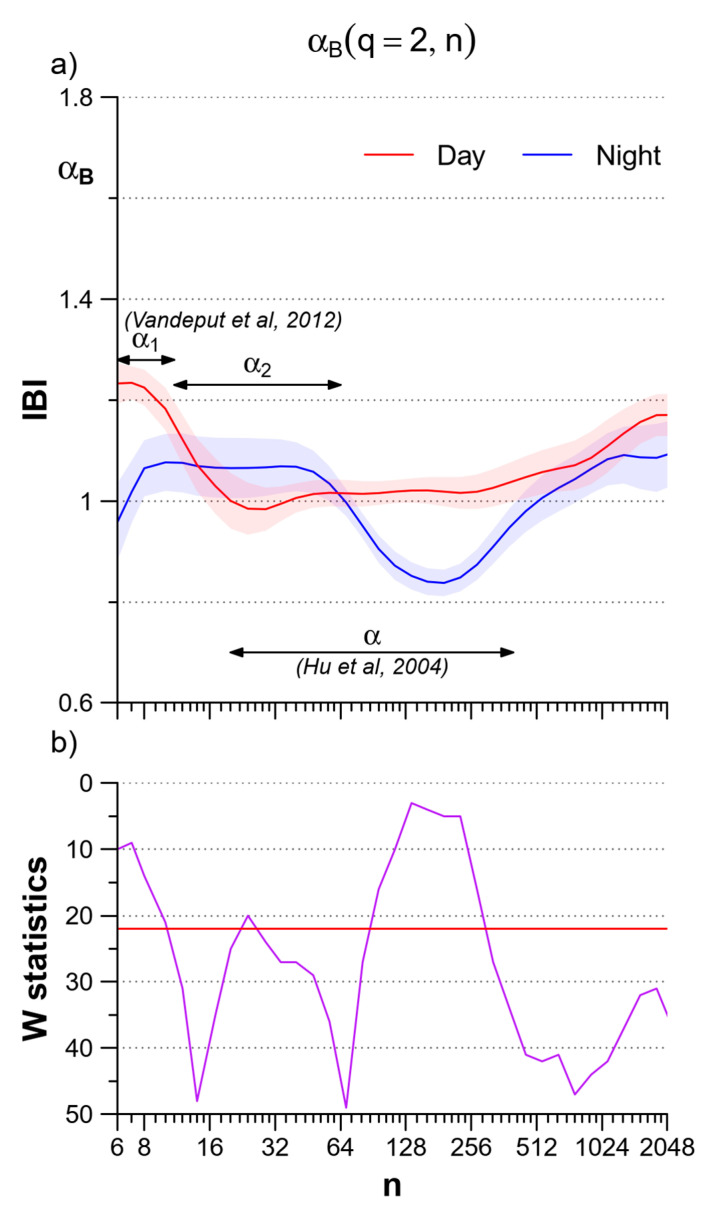
Day–night comparison of the multiscale monofractal DFA coefficients of IBI plotted vs. the block size *n* in beats. (**a**) *α*_B_(*q*,*n*) calculated for *q* = 2 (second-order moment of the monofractal DFA) during daytime (red) and nighttime (blue): mean +/− sem over the group of N = 14 participants; the arrows indicate the scale ranges for estimating *α*_1_ and *α*_2_ as defined by Vanderput et al. in [31] and for estimating *α* as defined by Hu et al. in [30]. (**b**) W statistics for the day-night difference in *α*_B_(2,*n*); when W is above the red horizontal line, the difference at the corresponding scale is significant at *p* < 5%.

Night–day modulations of the heart rate self-similarity coefficients were also reported in a study on 24-h Holter’s recordings performed on a large population of healthy subjects [30]. This study applied the bi-scale model as in [3], which originally defined a short-term coefficient α_1_ for scales between 4 and 16 beats and a long-term coefficient α_2_ for scales between 16 and 64 beats. The study in [30] used the scale *n* = 11 beats to separate α_1_ from α_2_ and reported a significant decrease in α_1_ at night. We did not consider scales short as in this study because at *τ* < 8 s the multifractal estimates can be affected by large estimation bias for negative *q* orders. However, α_1_ and the LF/HF powers ratio of the heart rate are correlated [13] and the reduction in the LF/HF powers ratio we reported at night in Table 1 is coherent with the night reduction of α_1_ in [30]. These authors, however, also showed a significant increase of α_2_ at night, which appears in contrast with the night decrease of the long-term scale coefficients reported both in [29] and in our work. To correctly interpret the results of the three studies, we should consider carefully the scale ranges where the coefficients are estimated. To illustrate this point, Figure 8 plots the coefficients we calculated as the derivative of log *F_q_*(*n*) vs. log *n* in Equation (1), i.e., α_B_(*q*,*n*), for *q* = 2. The scale *n* is expressed in beats to facilitate the comparison with previous studies [29,30]. As the estimation bias is negligible for *q* = 2, α_B_ is plotted from *n* = 6 beats. The night/day comparison shows a significant nocturnal decrease of α_B_ at scales < 11 beats, in line with the α_1_ results in [30], and greater night-time values at scales where α_2_ was estimated in [30]. These greater values correspond to the small area of statistical significance that appears in our Figure 3 at scales *τ* ≤ 16 s and at orders *q* ≥ 2. The α coefficient calculated in [29] between 20 and 400 beats overlaps partially with α_2_ but covers a much wider range of longer scales, which includes the band between 128 and 256 beats where we found a significant night decrease of α_B_. Therefore our study and the studies in [29,30] provide coherent results if the correct scale ranges are considered. The comparison of Figure 8 also highlights the importance to provide estimates of the scale coefficients as a continuous function of the scale *n* to correctly identify phenomena which may occur in nearby scales with different characteristics.

### 4.2. Nonlinearity

The comparison between original and Fourier-shuffled surrogates allowed us defining two concise indices of nonlinearity, *NL*_S_(*q*) and *NL*_L_(*q*), that indicate the moment orders and the scale ranges, where α(*q*,*τ*) provides information on nonlinear dynamics. These indices are close to 0% for *q* = 2, supporting previous theoretical speculations indicating that the monofractal DFA and the power spectrum provide similar information [12,13]. However, we also found clear nonlinear components for *q* between −2 and +4 at the short scales, more pronounced at night, both for the heart rate and the blood pressure. Furthermore, at night, substantial nonlinear components appear in heart rate at the longer scales. It should be noted that higher nonlinear components at night have been previously demonstrated by a noise titration procedure applied to Volterra/Wiener models fitting 24-h heart rate series [30]. The similarity of results obtained with so different approaches supports the evidence that nonlinearity prevails at night. 

The finding of important nonlinear components detected by the multifractal multiscale DFA method may help designing future clinical procedures aimed at better assessing the cardiovascular risk. Actually, a recent review of complexity-based methods for the analysis of heart rate variability reported that the prediction of cardiac events by the traditional short-term coefficient of the bi-scale monofractal DFA, α_1_, and by the standard spectral methods are correlated [31]. This inevitably reduces the additional prediction power of α_1_ compared to the spectral method. The traditional bi-scale monofractal model is based on the second-order moment, *q* = 2. By showing that DFA coefficients evaluated for *q* ≠ 2 provide information substantially different from that of the spectral powers, particularly at night, our study suggests that the multifractal multiscale DFA approach might effectively integrate the information of traditional spectral methods, possibly improving the clinical value of DFA.

Finally, an unexpected pattern in the Fourier-shuffled surrogate series of Figure 5 and Figure 6 consists in systematically higher α values for positive than for negative *q* orders (blue lines above red lines) when α increases with *τ* and in the opposite pattern (blue lines below red lines) when α decreases with *τ*. As a possible explanation of this pattern, we may hypothesize that cross-over scales appear anticipated at shorter scales when *q* > 0 and delayed at larger scales when *q* < 0.

## 5. Limitations and Conclusions

Nowadays, the clinical practice replaced the continuous invasive measures with intermittent noninvasive blood pressure measures for monitoring free-moving subjects, limiting the number of recordings available for the present study. Thus, it was not possible to stratify our results by gender or age, factors possibly influencing the circadian profile of the cardiovascular complexity [30]. Future studies on cardiovascular complexity can make use of noninvasive instrumentation measuring arterial blood pressure at the finger site continuously for 24 h even in ambulant subjects [32]. However, the scale coefficients of SBP could be affected by the amplification of the Mayer waves when blood pressure is measured at the digital artery [24]. Furthermore, if IBI is derived as the series of intervals between consecutive R peaks of the electrocardiogram rather than between consecutive pulses of blood pressure, as in this study, results at the shortest scales might differ because of the different amplitude of the respiratory sinus arrhythmia [33].

Finally, this study represents the temporal scales in seconds of time and not in number of beats, a relatively new methodological aspect originally proposed for comparing conditions with markedly different heart rate levels after selective autonomic blockade [6]. We adopted the same approach here because of the day–night differences in the mean heart rate and because we expected the differences to involve neural/humoral mechanisms which depend on time delays in seconds and not in number of beats (let’s think to the Mayer rhythm with a 10-s period due to the slow response of vascular resistances; or to the dynamics of removal of noradrenaline released by the sympathetic nerve endings, with a time constant of 1 min; or to long-term humoral fluctuations possibly responsible for the circadian component we found at scales of about 4 min). Mapping the temporal scales from beats *n* to time *τ* does not change the estimate of α, still based on the calculation of the derivative of log *F_q_*(*n*) vs. log *n*. This axis transformation is similar to mapping the “cycles/beat” in “Equivalent Hz” in the spectral analysis of cardiovascular series [34]. However, if results obtained with scales expressed as *τ* in seconds are discussed in relation to other studies based on scales defined in number of beats, readers should be aware that discrepancies may arise because possible differences in the heart rate level between conditions or groups may change the ranges of scales that define short-term and long-term DFA coefficients.

In conclusion, the multifractal multiscale DFA provides a detailed description of the complexity features of the cardiovascular series and highlights circadian modulations occurring at specific scales and affecting the individual fractal components differently. In perspective, by focusing on the more informative portions of the α(*q*,*τ*) surface it could be possible to design more powerful tools for assessing the cardiovascular risk. Furthermore, coefficients with *q* ≠ 2 reflect well the nonlinear components during night-time sleep, suggesting that they may effectively integrate the spectral information with complexity-domain information. Therefore, the evaluation of the multifractal multiscale surface of scale coefficients during wake and sleep may improve the risk assessment in cardiovascular prevention, the evaluation of cardiovascular interventions as well as the monitoring of the efficacy of rehabilitation protocols.

## Figures and Tables

**Table 1:** Mean levels and spectral powers of cardiovascular series.

	Day	Night	*p* Value
**IBI**			
*mean* (ms)	774.4 (97.3)	1033.7 (174.1)	<0.01
*total power* (ms^2^)	11,217 (10,569)	11,751 (7313)	0.57
*VLF power* (ms^2^)	5885 (5763)	5905 (3599)	0.62
*LF power* (ms^2^)	1453 (1219)	2083 (1946)	0.25
*HF power* (ms^2^)	538 (576)	1219 (1036)	<0.01
*LF/HF powers ratio*	3.56 (1.4)	2.21 (1.5)	<0.01
**SBP**			
*mean* (mmHg)	123.7 (12.8)	108.6 (17.5)	<0.01
*total power* (mmHg^2^)	134.7 (98)	58.4 (35.4)	<0.01
*VLF power* (mmHg^2^)	65.0 (49.9)	29.3 (19.4)	<0.01
*LF power* (mmHg^2^)	22.8 (13.4)	9.7 (6.2)	<0.01
*HF power* (mmHg^2^)	7.3 (4)	4.0 (2.3)	<0.01
**DBP**			
*mean* (mmHg)	70.2 (8.9)	60.2 (10.1)	<0.01
*total power* (mmHg^2^)	53.5 (22.6)	30.4 (17.9)	<0.01
*VLF power* (mmHg^2^)	25.8 (12.7)	15.3 (9.5)	<0.01
*LF power* (mmHg^2^)	10.3 (4)	5.6 (3.5)	<0.01
*HF power* (mmHg^2^)	2.8 (1.1)	1.8 (1.1)	<0.01

Values as mean (SD); *p* value after *T* test on log-transformed powers.
